# The Microscopist's Companion

**Published:** 1859-07

**Authors:** 


### BIBLIOGRAPHICAL.

*The Microscopist's Companion*, a popular Manual of Practical Microscopy, to which is added a *Glossary* of the principal terms used in Microscopic Science. By John King, M. D., 8vo. pp. 304, Cincinnati: Robert Clarke & Co., 1859.

With the views held by this Journal upon Microscopic Science, we cannot but hail with pleasure every effort promoting or tending to promote its advancement, the more especially when that effort is made by our own countrymen, be they authors or scientific artists. Our people are too prone to overlook the rich profusion of material at home, and to undervalue indigenous talent which shall attempt to interpret the great truths that it may lay bare, while they look in full faith towards oracular Europe; but at this time it were gross ignorance not to know what has been done by such men as Bailey, Riddell and Edwards, with that unrivalled instrument the American microscope.

We therefore welcome the volume before us because it is American; but we feel compelled to withhold from it unmeasured praise, notwithstanding. The book is divided into two parts. The first, comprising about two hundred pages, treats of the microscope, its use and application, and the examination and preservation of objects usually known as microscopic: the second consists of a very ingenious glossary, of a hundred pages. On the whole it is a clever production, but unfortunately we see too little of the author in it, although we cannot but commend the good sense which has guided his compilations. The work is illustrated by 114 wood cuts, which are fairly executed; but we beg leave to suggest that in a future edition, the number of illustrations be increased, so as to give the less advanced student more representations, proportionately to the whole, of what the mind is exercised upon through the microscope, than of the instrument itself under every respect and in all its details. We would like to find more attention paid to the faithful and artistic delineation of the microscopic image of the microscopic object; to have, in connection with test objects a figure of some easily attainable valve, for instance, as resolved by and associated with each objective; and to have represented that variety only of forms of the microscope as may illustrate principle and necessity in the diversity. And we would like above all in a new book, to have original illustrations.

We regret that the subject of Preparation of Sections should be so cursorily treated, and especially the hard tissues and substances, for we hold no details nor any good method superfluous in a "Microscopist's Companion," seeing that in this country, where the field is so fraught with novelty and interest, a successful Microscopist must be his own preparer of specimens, which often must be numerous and serially arranged. Besides the expense, uncertainty and delay attendant upon procuring European mountings of American objects, the observer purchasing such objects is restricted in the choice of specimens, or else cannot choose at all. We therefore expect to be furnished with the most complete and satisfactory information upon these very practical points; and we are certain that dentists prosecuting science, will feel this omission very sensibly.

Under the caption "Methods of Measurement," are to be found the usual indications for determining the *power* of any combination of an eye-piece and an objective; but it has occurred to us that it is not possible to fix absolutely the linear amplification of a combination, unless we can assume as established a precise distance of ordinary distinct vision, which varies not only in different individuals, but has also its far and near limits by virtue of the power of accommodation of the eye. Consequently if we regard ten inches as approximative of that distance, we can arrive at an approximation only of the power of an ocular with its objective, which is good no longer than the unengaged eye measures the scale under the same visual angle. Besides, as G. Jackson observes, we cannot have entire confidence of the *extreme* accuracy of such a method, for when the divisions of the scale are minute it is very difficult to observe their *exact coincidence* with the image of the object to be measured, when the slightest movement of the head must alter their relative position.

We are disposed to regard the affixing of *prices* to the microscopes, accessories, &c., with favor, although Tolles' list does not appear otherwise than as an advertisement at the end of the book.

To recapitulate, we recognize in the "Companion" a very useful and instructive little work, notwithstanding our objections; and we cheerfully give credit to its author for his industry as an observer, as may be witnessed for example, on pages 108 and 109.

We have no idea of the commercial value of the book, although we presume that it will take its place alongside of works on the Microscope, like Beale's, Wyeth's and Jabez Hogg's.

### EDITORIAL DEPARTMENT.

